# Tumor Seeding of Percutaneous Nephrostomy Tract from Urothelial Carcinoma of the Kidney

**DOI:** 10.1155/2013/819470

**Published:** 2013-10-30

**Authors:** Igor Sorokin, R. C. Welliver, Osama Elkadi, Tipu Nazeer, Ronald P. Kaufman

**Affiliations:** ^1^Division of Urology, Albany Medical College, Albany, NY 12208, USA; ^2^Department of Pathology, Albany Medical College, Albany, NY 12208, USA; ^3^Urological Institute of Northeastern New York, Albany, NY 12208, USA

## Abstract

Urothelial carcinoma (UC) of the renal pelvis has been rarely shown to metastasize to the skin. Tumor seeding from iatrogenic procedures is a source of spreading of UC to the skin. We herein present a case of primary UC of the renal pelvis with spreading to the skin from a percutaneous nephrostomy tract.

## 1. Introduction

The most common sites of metastatic disease from urologic malignancies include the regional lymph nodes, liver, lung, and bone [1]. Urothelial carcinoma (UC) of the genitourinary system rarely metastasizes to the skin. The incidence is reported to be less than 1%, with most cases reported from the urinary bladder [2, 3]. Rarely, iatrogenic procedures have been identified as the cause of seeding of tumor to the skin [4]. We present a rare case of renal pelvic UC where tumor seeding occurred along a percutaneous nephrostomy tract to the skin. 

## 2. Case Report

A 76-year-old male presented with a history of solitary right kidney after a left nephrectomy for a nonfunctional kidney. He developed low grade stage Ta UC in the right renal pelvis that was initially diagnosed in 2002 with multiple recurrences as well as migration to the ureter and bladder. This was previously managed with endoscopic fulguration of the tumor periodically as he was inconsistent in his followup. He then presented in June 2012 with acute renal failure due to ureteral obstruction from the tumor. A percutaneous nephrostomy (PCN) was placed emergently by interventional radiology. He underwent an endoscopic procedure in September 2012, but due to large tumor volume he was unable to be completely treated at that last visit. His PCN was kept in place to attempt percutaneous management of his tumor at his next visit.

The patient was taken back to the operating room in November 2012 for the planned percutaneous fulguration. The case was started from a retrograde ureteroscopic approach to ablate the portions of the kidney that we felt would be easier managed ureteroscopically. We had fulgurated portions of the mid and lower pole when anesthesia staff noted that the patient was tachycardic and hypotensive (60s/40s), and the case was stopped to resuscitate the patient. During the evaluation, his PCN was pulled out and was found on the operating room floor. After the patient became more stable, we used a flexible cystoscope to place a right ureteral stent under fluoroscopic guidance. We were never able to commence the percutaneous portion of the procedure, and the patient was transferred to the surgical intensive care unit. He recovered from his urosepsis without sequelae and was able to be discharged home. Histopathologic evaluation of the upper pole lesion revealed a high grade urothelial carcinoma involving the collecting ducts.

**Figure 1:**
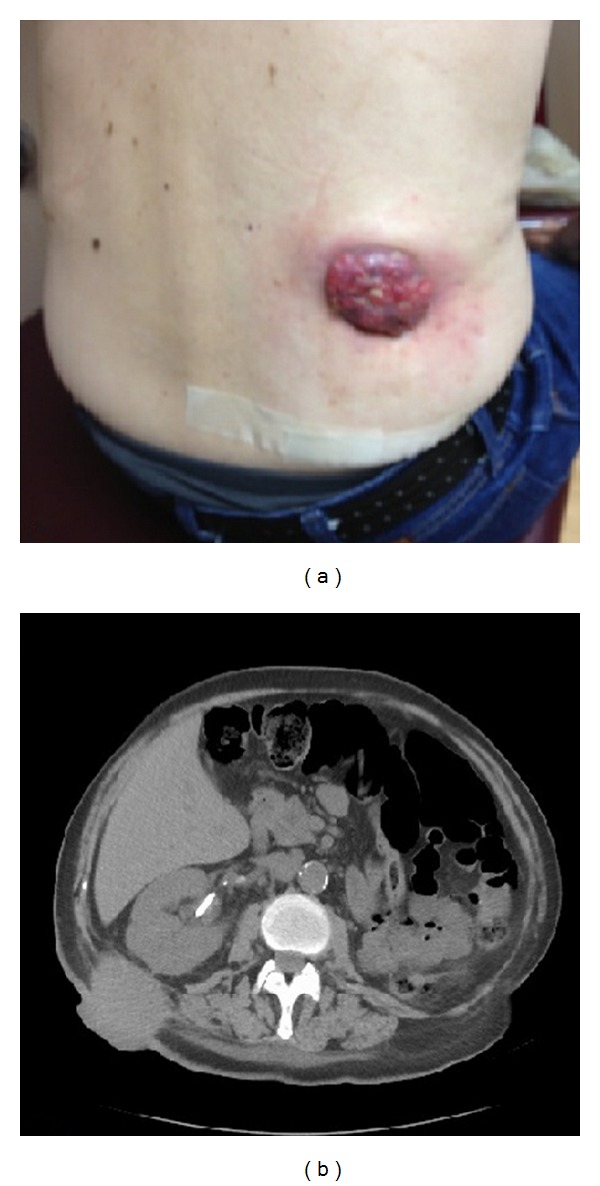
(a) Soft tissue mass at previous traumatically removed nephrostomy site, (b) CT scan revealing extent of the soft tissue mass.

**Figure 2:**
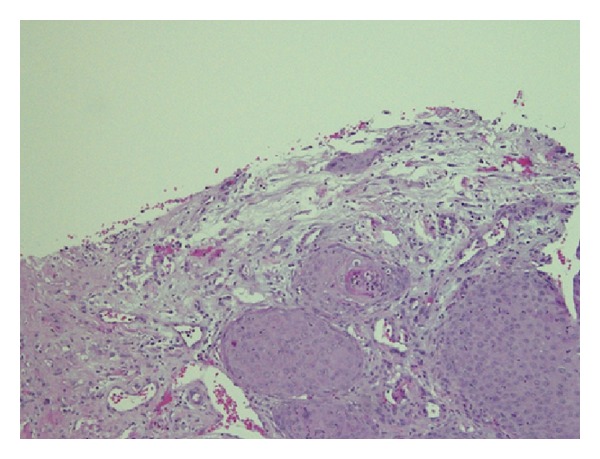
Biopsy of soft tissue mass which revealed high grade urothelial carcinoma with squamous features.

**Figure 3:**
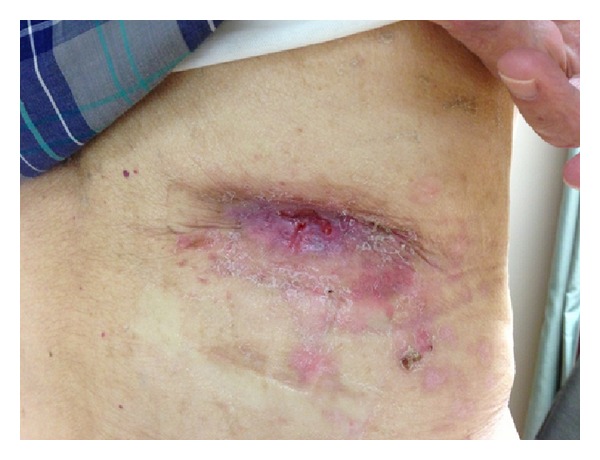
Resolution of mass after chemotherapy and radiation treatment.

 At his follow-up visit a few weeks later, the patient was noted to have a soft tissue mass at the previous percutaneous nephrostomy tract site (Figure 1(a)). He also underwent a computed tomography (CT) scan which revealed the soft tissue mass that was found (Figure 1(b)). This was biopsied and showed an infiltrating high grade carcinoma with squamous features, including intercellular bridges and focal keratinization (Figure 2). This tumor bears some morphologic similarities to the patient's previously diagnosed urothelial carcinoma. He received chemotherapy and radiation on his flank for the mass which eradicated the tumor from the outside (Figure 3). The patient ultimately expired a few months later from metastatic disease. 

## 3. Discussion

Cutaneous metastasis of UC is an extremely rare condition, especially in the case of UC of the renal pelvis [3, 5]. It is generally accepted as a late manifestation of systemic disease [6]. One case report diagnosed metastatic UC in a woman with bilateral arm nodules as her systemic recurrence of the disease 1.5 years after a previous nephroureterectomy; her disease was previously limited to her renal pelvis. Another case report described metastatic UC of the renal pelvis manifesting as an ulcerative lesion of the penis after nephroureterectomy performed 8 years before. The rate of skin metastasis from UC of the renal pelvis is currently unknown and likely very rare but does carry a very poor prognosis [6]. 

Iatrogenic procedures have been known to cause spreading of tumor to the skin, even in the face of low grade UC. Procedures such as partial cystectomy, suprapubic cystotomy, pyelotomy, and laparoscopy have all been reported to cause seeding of tumor to the skin [4, 7]. There are also reports of tumor seeding of renal cell carcinoma along probe tracts used for cryoablation and diagnostic biopsies [8]. 

Squamous differentiation occurs in about 20% of urothelial carcinoma of the bladder with varying proportions. The frequency of squamous differentiation increases with grade and stage. It has been reported that these tumors have less favorable response to therapy than pure urothelial carcinoma [9]. Since the patient's primary urothelial cancer was not completely resected before, the presence of squamous differentiation could not be confirmed in his primary tumor. However, frank squamous differentiation was demonstrated in his metastatic tumor in the right flank.

In our case, there were likely 2 main causes of tumor seeding. The first is the duration of the PCN for 5 months. The second is the inadequate and insufficient resection of his extensive tumor which was limited by his deterioration in the OR. Our case illustrates that awareness of this rare complication is particularly relevant to those with multiple recurrences of UC managed with PCN. This case illustrates the importance of limiting the duration of use of PCN in these patients. Additionally, this case shows cause for resection of nephrostomy tract in patients with a PCN in place for an extended period of time.
